# Neo-synthesis of estrogenic or androgenic neurosteroids determine whether long-term potentiation or depression is induced in hippocampus of male rat

**DOI:** 10.3389/fncel.2015.00376

**Published:** 2015-10-02

**Authors:** Michela Di Mauro, Alessandro Tozzi, Paolo Calabresi, Vito Enrico Pettorossi, Silvarosa Grassi

**Affiliations:** ^1^Dipartimento di Medicina Sperimentale, Sezione di Fisiologia e Biochimica, Università di PerugiaPerugia, Italy; ^2^Fondazione Santa Lucia – I.R.C.C.S.Roma, Italy; ^3^Dipartimento di Medicina, Clinica Neurologica, Università di PerugiaPerugia, Italy

**Keywords:** 17β-estradiol, 5α-dihydrotestosterone, P450-aromatase, 5α-reductase, hippocampus, long-term potentiation, long-term depression, depotentiation

## Abstract

Estrogenic and androgenic steroids synthesized in the brain may rapidly modulate synaptic plasticity interacting with specific membrane receptors. We explored by electrophysiological recordings in hippocampal slices of male rat the influence of 17β-estradiol (E2) and 5α-dihydrotestosterone (DHT) neo-synthesis on the synaptic changes induced in the CA1 region. Induction of long-term depression (LTD) and depotentiation (DP) by low frequency stimulation (LFS, 15 min-1 Hz) and of long-term potentiation (LTP) by high frequency stimulation (HFS, 1 s-100 Hz), medium (MFS, 1 s-50 Hz), or weak (WFS, 1 s-25 Hz) frequency stimulation was assayed under inhibitors of enzymes converting testosterone (T) into DHT (5α-reductase) and T into E2 (P450-aromatase). We found that LFS-LTD depends on DHT synthesis, since it was fully prevented under finasteride, an inhibitor of DHT synthesis, and rescued by exogenous DHT, while the E2 synthesis was not involved. Conversely, the full development of HFS-LTP requires the synthesis of E2, as demonstrated by the LTP reduction observed under letrozole, an inhibitor of E2 synthesis, and its full rescue by exogenous E2. For intermediate stimulation protocols DHT, but not E2 synthesis, was involved in the production of a small LTP induced by WFS, while the E2 synthesis was required for the MFS-dependent LTP. Under the combined block of DHT and E2 synthesis all stimulation frequencies induced partial LTP. Overall, these results indicate that DHT is required for converting the partial LTP into LTD whereas E2 is needed for the full expression of LTP, evidencing a key role of the neo-synthesis of sex neurosteroids in determining the direction of synaptic long-term effects.

## Introduction

Synaptic long-term potentiation (LTP) and long-term depression (LTD) are usually induced in the hippocampus by high frequency stimulation (HFS) and low frequency stimulation (LFS), respectively, and are commonly regarded as the cellular substrate for learning and memory (Bliss and Lomo, 1973; Staubli and Lynch, 1990; Bliss and Collingridge, 1993; Dudek and Bear, 1993; Bear and Malenka, 1994; Staubli and Ji, 1996; Martin et al., 2000). LTP and LTD are considered models of bidirectionally-changeable plasticity that are generally dependent on NMDA receptor (NMDAR) activation followed by postsynaptic Ca^2+^ influx, able to trigger Ca^2+^-dependent signaling pathways in many brain regions such as the hippocampus, in particular at the level of the Schaffer collateral-CA1 synapse (Bliss and Collingridge, 1993; Malenka and Nicoll, 1999; Malenka and Bear, 2004). Therefore, the direction of the change of synaptic strength depends on afferent stimulation causing different activation of NMDAR-dependent Ca^2+^ signaling (Dudek and Bear, 1992, 1993; Mulkey and Malenka, 1992; Bear and Malenka, 1994; Cummings et al., 1996). Thus, a larger HFS-dependent Ca^2+^ entry leads to LTP (Bliss and Collingridge, 1993; Bear and Malenka, 1994), while moderate Ca^2+^ influx caused by LFS induces LTD in naïve synapses or de-potentiation (DP) in potentiated synapses (Staubli and Lynch, 1990; Dudek and Bear, 1992, 1993; Bear and Malenka, 1994; Staubli and Ji, 1996).

Growing evidence suggest that the sex steroids 17β-estradiol (E2), testosterone (T) and 5α-dihydrotestosterone (DHT) may participate in rapidly modulating the long-term synaptic effects in different areas of the brain (McEwen, 2002; Isgor and Sengelaub, 2003; MacLusky et al., 2006; Hajszan et al., 2008) interacting with membrane receptors for E2 (ERs) and androgens (ARs; Kerr et al., 1995; Milner et al., 2001, 2005; Kalita et al., 2005; Tabori et al., 2005; Pedram et al., 2006; Foradori et al., 2008; Morissette et al., 2008; Raz et al., 2008; Levin, 2009). In particular, it has been shown that E2 can increase the NMDAR-mediated glutamatergic transmission, decrease the GABAergic one and enhance the magnitude of LTP at hippocampal CA3-CA1 glutamatergic synapses (Wong and Moss, 1992; Woolley et al., 1997; Murphy et al., 1998; Foy et al., 1999; Foy, 2001; Rudick and Wooley, 2001; Smith and McMahon, 2005, 2006; Smith et al., 2009; Hasegawa et al., 2015), while T and DHT show an opposite effect (Harley et al., 2000; Hebbard et al., 2003; Skucas et al., 2013; Hasegawa et al., 2015).

We recently reported that in hippocampal slices of male rats, estrogenic and androgenic signals are selectively involved in the induction of LTP or LTD/DP in response to specific synaptic activation (Pettorossi et al., 2013). Moreover, it has been found, by blocking ERs and ARs that E2 is implied in the LTP induced by HFS, while androgens in LTD/DP induced by LFS. This opposite influence of sex steroids on synaptic plasticity could be exerted by either the circulating steroids of gonadal origin or steroids synthesized in the nervous system from cholesterol (Baulieu, 1997; Compagnone and Mellon, 2000) and the subsequent conversion of T into E2 and DHT by P450-aromatase and 5α-reductase enzymes, respectively (Selmanoff et al., 1977; Kimoto et al., 2001; Hojo et al., 2004, 2008, 2009; Mukai et al., 2006). It is important to distinguish the specific influence of neurosteroids synthetized *de-novo* within the central nervous system (CNS) from that of circulating steroids since the neo-synthesis may directly reflect the functional conditions of CNS and may vary depending on the neuronal activity *per se* (Kimoto et al., 2001; Hojo et al., 2004, 2008, 2009; Balthazart and Ball, 2006; Balthazart et al., 2006; Mukai et al., 2006; Ooishi et al., 2012). The relevance of the sex neurosteroid synthesis is evidenced by their concentration in the nervous system that is significantly higher than that in the circulatory system (Selmanoff et al., 1977; Kimoto et al., 2001; Hojo et al., 2004, 2008, 2009; Mukai et al., 2006).

We previously reported that HFS-LTP is markedly reduced by the blocking agent for the P450-aromatase activity, letrozole (Grassi et al., 2009, 2011; Tanaka and Sokabe, 2012; Vierk et al., 2012, 2014), supporting the involvement of the E2 neo-synthesized within the CNS in the induction of LTP. However, the possible contribution of neo-synthesis of E2 in LTP and LTD induced by different stimulation patterns, or the role of the neo-synthesis of androgens in LTP and LTD has not been addressed.

Therefore, in the present study we directly assessed, in the hippocampal slices of male rat, the role of E2 and DHT neo-syntheses and their possible interaction in the induction of LTD/DP and LTP focusing on the Schaffer collateral-CA1 synaptic region where the LTP is known to be NMDAR dependent. For this purpose, we analyzed the effect of different stimulation patterns in the presence of inhibitors of the P450-aromatase and/or the 5α-reductase enzymes.

## Material and Methods

### Ethic Statement on Animal Use

All procedures on animals were conducted in conformity with the guidelines of the Italian Ministry of Health, national laws on animal research (Legislative Decree 26/2014) and European Communities Council Directive (86/609/ECC), in accordance with protocols approved by the Animal Care and Use Committee at the University of Perugia (Italy). Wistar rats (Harlan, Italy) (2 per cage) were kept under regular lighting conditions (12 h light/dark cycle) and given food and water *ad libitum*. All efforts were made to minimize the number of animals used and their suffering.

### Electrophysiology

The study was conducted in 281 hippocampal slices prepared from 112 male Wistar rats at P50–60. We used male rats to avoid any possible influence of cyclic, systemic estrogenic fluctuation on the induction of synaptic plasticity (Warren et al., 1995; Good et al., 1999). Animals were sacrificed under deep halothane anesthesia, by cervical dislocation. The brain was rapidly removed and immersed for 2–3 min in ice-cold ACSF containing (in mM): 126 NaCl, 2.5 KCl, 1.2 MgCl_2_, 1.2 NaH_2_PO_4_, 2.4 CaCl_2_, 10 glucose, and 25 NaHCO_3_, continuously bubbled with 95% O_2_ and 5% CO_2_, pH 7.4. After the extraction of the hippocampus, 400 μm-thick transverse slices were cut in ice-cold ACSF with a vibratome (Series 1000 plus starter CE, Vibratome, St. Louis, MO, USA) and allowed to recover in oxygenated ACSF at room temperature for 2 h before experimental recordings.

### Field Potential Recordings

For each animal we used 2–3 slices. A slice was transferred into the recording chamber and submerged with ACSF at a constant rate of 2 ml/min at room temperature.

Extracellular recordings with borosilicate glass capillaries (GC150F-10; Harvard Apparatus) filled with 2M NaCl (resistance, 10–15 MΩ) were obtained from the apical dendritic layer of the CA1 region for analysis of population EPSPs. Synaptic responses were elicited by applying single stimuli pulses (duration: 20 μs and intensity: 20–50 mA) at a frequency of 0.05 Hz through a bipolar platinum-iridium stimulating electrode placed in the Schaffer collateral–commissural pathway. This stimulation evoked field EPSPs (fEPSPs) that were 50–70% of maximal slope. FEPSPs were filtered at 3 KHz, digitized at 10 KHz and stored on PC equipped with a data acquisition card (at-MIO-16E-2, National Instruments, Austin, TX, USA). An Axoclamp 2B amplifier (Molecular Devices, USA) was used for the recordings.

After a stable baseline recording for 20 min, LTD/DP or LTP was induced. For inducing LTD and DP we used a LFS protocol consisting of 15 min stimulation at 1 Hz applied at the same stimulus intensity. LTP was normally induced by HFS (a single 1 s-100 Hz tetanus) at the same stimulus intensity. In some experiments the LTP induction was investigated by using a single weak frequency stimulation (WFS, 1 s-25 Hz tetanus) or medium frequency stimulation (MFS, 1 s-50 Hz tetanus).

### Drugs

E2 (0.5–1 nM), DHT (10–50 nM), T (50 nM), the specific inhibitor of the enzyme 5α-reductase finasteride (1 μM) (Finn et al., 2006) and the specific inhibitor of P450-aromatase enzyme letrozole (100 nM) (Bhatnagar et al., 2001) were used for the experiments. All drugs were purchased from Sigma-Aldrich (St Louis, MO, USA). Stock drug solutions were dissolved in DMSO, diluted to working concentration in oxygenated ACSF and perfused at a rate of 2 ml/min. Total replacement of the medium in the chamber occurred within 1 min. In the experiments in which the effect of LFS, HFS, WFS or MLF was analyzed in the presence of blocking agents, drugs were applied for all the recording period 15 min before the application of the stimulation protocol. The influence of drug vehicle (0.001% DMSO) on the induction of LTD/DP and LTP was excluded on the basis of analysis performed previously (Pettorossi et al., 2013).

### Electrophysiological Data Analysis and Statistical Evaluation

To characterize the drug effects on the baseline fEPSP and on the induction of the long-term effects caused by different stimulation protocols, testing stimuli were applied every 20 s. The initial slope of fEPSP was measured using linear regression of the first 0.8 ms succeeding the pre-synaptic fiber volley and the average response recorded during a stable period (10 min) at the beginning of the experiment was used as the baseline. The averaged fEPSP, calculated every 2 min, was expressed as percentage of the baseline fEPSP value and used for data presentation. In each experiment, the occurrence of LTD or LTP was statistically verified (Student’s paired *t* test) by comparing the fEPSP slopes measured 40 min following the inducing stimulus relative to baseline responses. To prove the induction of DP (Staubli and Lynch, 1990; Dudek and Bear, 1993) we compared in each experiment (Student’s paired *t* test) the pre-LFS fEPSP values with those measured 40 min after LFS. In addition, the effects of drugs on the baseline were evaluated by comparing (Student’s paired *t* test) the pre-drug fEPSP values with those measured 10–15 min after the drug application. Moreover, the effects observed in different experimental conditions were compared by using the one-way analysis of variance (ANOVA) and the Tukey’s *post hoc* test. The level of significance was set at *p* < 0.05 for Student’s *t* test, ANOVA and *post hoc* comparisons. Statistical analyses were performed with Statistica (StatSoft, Tulsa, OK, USA). Values given in the text are mean ± SEM, *n* representing the number of the slices.

## Results

### Role of DHT and E2 Neo-Syntheses in the Development of LFS-LTD

#### Inhibition of 5α-Reductase by Finasteride Prevents the Development of LFS-LTD

**Figure 1:**
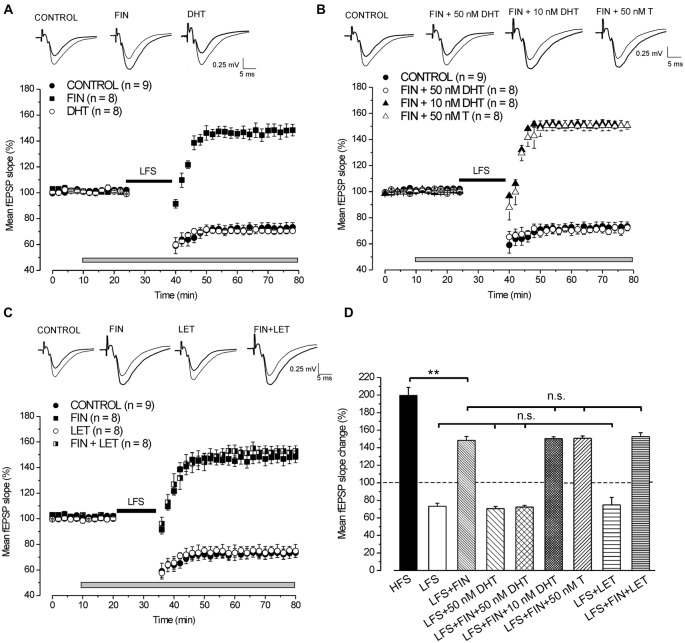
**Induction of long-term depression (LTD) by low frequency stimulation (LFS) depends on the synthesis of DHT. (A,C)** Effects of LFS in the presence of different drugs. On the top of panels **(A–C)** averaged traces (*n* = 20) of fEPSPs recorded before (thin traces) and 40 min after LFS (thick traces) in different experimental conditions. Graphs in this and following figures report mean ± SEM (*n* = number of slices) of the fEPSP slope evaluated within 2-min interval and expressed as a percentage of the baseline. The gray bars show the drug infusion time and the black bars the LFS delivering time. **(A)** Effect of LFS in control condition (filled circles), under finasteride (FIN, filled squares) and 50 nM DHT (open circles). **(B)** Effect of LFS in control condition (filled circles), under FIN + 50 nM DHT (open circles), FIN + 10 nM DHT (filled triangles) and FIN + 50 nM T (open triangles).** (C)** Effect of LFS in control condition (filled circles), under FIN (filled squares), letrozole (LET, open circles) and FIN + LET (half-filled squares). **(D)** Comparison of the fEPSP changes induced by high frequency stimulation (HFS) in control condition and by LFS under different drugs as shown in **(A–C)**. In this and following bar charts data represent mean ± SEM of the fEPSP slope (% of the baseline). Comparisons among long-term potentiation (LTP) (one-way analysis of variance (ANOVA), *F*_8,72_ = 54.2, *p* < 0.0001; Tukey’s *post hoc* test, **p < 0.001; n.s. = no significant) and LTD (one-way ANOVA, *F*_3,29_ = 0.2, *p* = 0.85). Note that without synthesis of DHT, LFS is able to induce a partial LTP that is not dependent on the E2 synthesis.

In control condition LFS induced LTD of synaptic transmission reducing the fEPSP to 73.3 ± 3.4% (*n* = 9, 4 animals, Figures 1A,D). The application of finasteride did not change the baseline (pre-drug 100.6 ± 0.6% vs. post-drug 101.1 ± 0.4%, *n* = 8, 3 animals, Student’s *t* test, *p* = 0.51, Figure 1A), but it fully prevented LFS-LTD. A partial LTP was induced instead, since LFS enhanced the fEPSP to 148 ± 4.5% (*n* = 8, Figures 1A,D). This potentiation was significantly smaller than the LTP normally induced by HFS (Tukey’s *post hoc* test, LFS + FIN vs. HFS control: *p* < 0.001, Figure 1D). Moreover, finasteride applied following LFS-LTD induction had no effect (pre-drug 73.1 ± 4.6% vs. post-drug 73.7 ± 4.3%, *n* = 4, 2 animals, Student’s *t* test, *p* = 0.39, data not shown).

#### Exogenous DHT Rescues the LFS-LTD under 5α-Reductase Inhibition by Finasteride

Application of 50 nM DHT did not modify either the baseline (pre-drug 100.7 ± 0.5% vs. post-drug 99.7 ± 0.2%, *n* = 8, 3 animals, Student’s *t* test, *p* = 0.18, Figure 1A) or the development of LFS-LTD (70.5 ± 2.5%, *n* = 8, Tukey’s *post hoc* test, LFS + 50 nM DHT vs. LFS control: *p* = 0.99, Figures 1A,D), but it was able to fully rescue the LFS-LTD under finasteride (72.3 ± 2%, *n* = 8, 3 animals, Tukey’s *post hoc* test, LFS + FIN + 50 nM DHT vs. LFS control, *p* = 0.99, Figures 1B,D). Conversely, this rescue did not occurr using lower concentration of DHT (10 nM) since LFS still induced a partial LTP (150.5 ± 2.1%, *n* = 8, 3 animals, Figure 1B, Tukey’s *post hoc* test, LFS + FIN +10 nM DHT vs. LFS + FIN, *p* = 0.99, Figure 1D). In addition, we also applied 50 nM testosterone (T) under finasteride for rescuing LTD. T did not change the baseline (pre-drug 100.7 ± 0.4% vs. post-drug 100.6 ± 0.1%, *n* = 8, 3 animals, Student’s *t* test, *p* = 0.87, Figure 1B) and was not able to rescue the LFS-LTD since a partial LTP was induced, not different from that observed under finasteride alone (150.7 ± 2.8%, *n* = 8, Tukey’s *post hoc* test, LFS + FIN +50 nM T vs. LFS + FIN: *p* = 0.99, Figures 1B,D).

Overall, these results on blockade of 5α-reductase provide evidence that the DHT synthesized *de-novo* plays a crucial role in the development of LFS-LTD, while T has no effect.

#### Inhibition of P450-Aromatase by Letrozole does not Affect the Development of LFS-LTD

Letrozole did not affect either the baseline (pre-drug 100.2 ± 0.2% vs. post-drug 100.7 ± 0.4%, *n* = 8, 3 animals, Student’s *t* test, *p* = 0.43, Figure 1C) or the development of LFS-LTD (74.8 ± 5%, *n* = 8, Tukey’s *post hoc* test, LFS + LET vs. LFS control, *p* = 0.99, Figures 1C,D). In addition, the partial LTP observed under fiansteride was not modified when LFS was delivered under letrozole plus finasteride (152.7 ± 4.3%, *n* = 8, 3 animals, Tukey’s *post hoc* test, LFS + FIN + LET vs. LFS + FIN, *p* = 0.98, Figures 1C,D). These data suggest that E2 neo-synthesis is not implied in the development of either LFS-LTD or LFS-small LTP.

#### Exogenous E2 Reverts LFS-LTD into LFS-LTP

**Figure 2:**
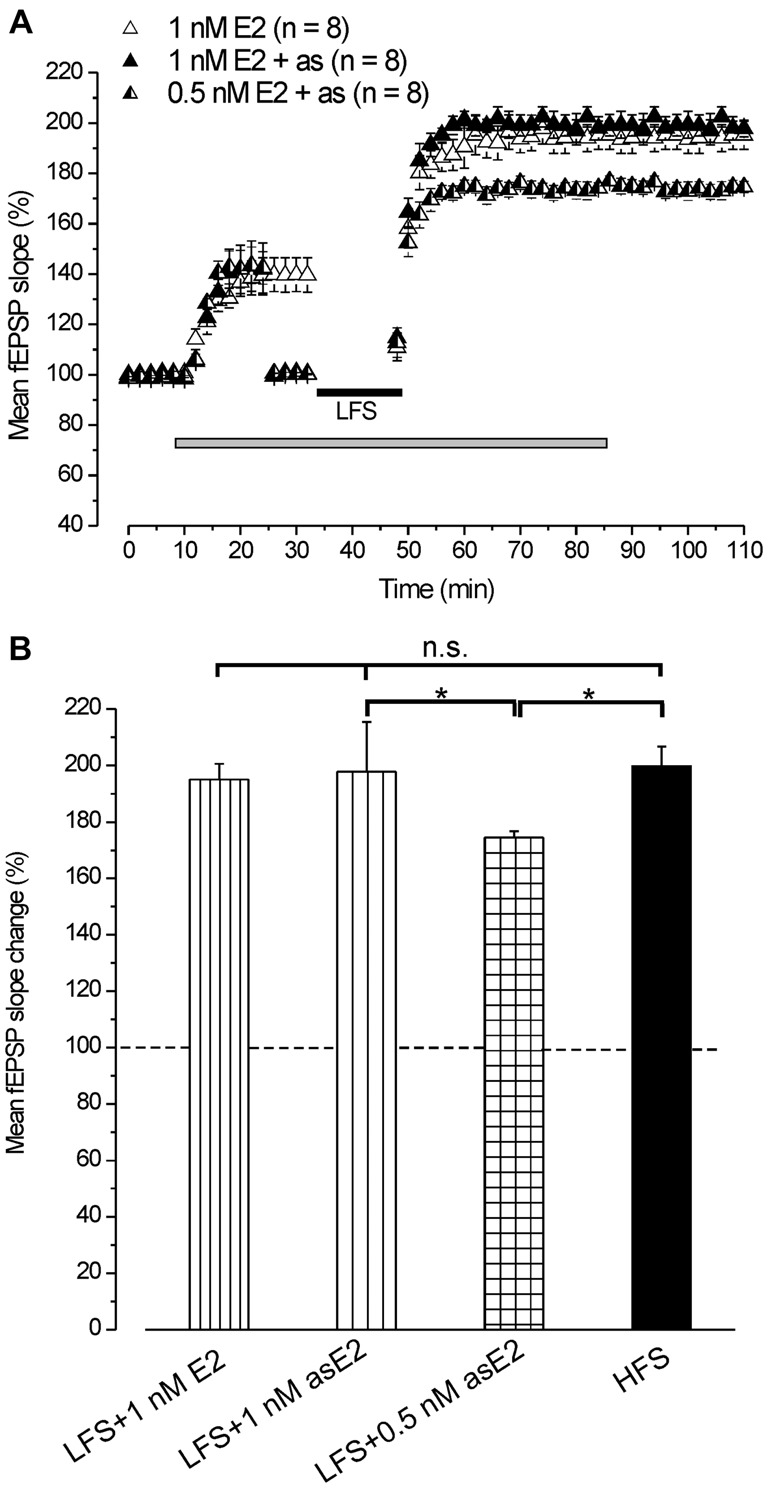
**Exogenous administration of E2 reverts the LFS-LTD into LFS-LTP. (A)** Effect of LFS in the presence of 1 nM E2 (open triangles) without stimulus adjustment and 1 nM (filled triangles) or 0.5 nM E2 (half-filled triangles) with stimulus adjustment (as). **(B)** Comparison among LTP induced by HFS in control condition and by LFS in the presence of E2 at different concentrations and stimulus adjustment (one-way ANOVA, *F*_3,37_ = 2.86, *p* < 0.05; Tukey’s *post hoc* test, *p < 0.05).

In agreement with earlier reports (Foy et al., 1999; Bi et al., 2001; Kramár et al., 2009), infusion of 1 nM E2 caused a rapid increase of the fEPSP baseline (139.4 ± 5.1%, *n* = 8, 4 animals, Figure 2A). Subsequent application of LFS elicited LTP (193.9 ± 5.3%, *n* = 8, Figure 2A) that persisted at the E2 washout (195.1 ± 5.5%, Student’s *t* test, *p* = 0.42) and was not different from the LTP induced by HFS (Tukey’s *post hoc* test, LFS + 1 nM E2 vs. HFS control, *p* = 0.97, Figure 2B). The same effect was observed when LFS was delivered under E2 after adjusting the stimulation pulse strength (asE2) to produce responses equivalent in size to those recorded during pre-infusion baseline (LTP: 197.8 ± 3.2%, *n* = 8, 3 animals, Tukey’s *post hoc* test, LFS + 1 nM asE2 vs. LFS + 1 nM E2, *p* = 0.87, LFS + 1 nM as E2 vs. HFS control, *p* = 0.99, Figures 2A,B). E2 used at lower concentration (0.5 nM) also increased the baseline (142.4 ± 3%, *n* = 8, 3 animals) similarly to what observed after 1 nM E2 (ANOVA: *F*_(1, 14)_ = 0.25; *p* = 0.68, Figure 2A). LFS delivered after adjusting the response to the pre-drug values induced LTP (174.5 ± 2.2%, *n* = 8, Figure 2A) that was lower than the one obtained in the presence of 1 nM asE2 (Tukey’s *post hoc* test: LFS + 0.5 nM asE2 vs. LFS + 1 nM asE2, *p* < 0.05, Figures 2A,B).

This result demonstrates that exogenously administered E2 is able to revert the LTD induced by LFS into LTP.

### Role of E2 and DHT Neo-Synthesis in the Development of LTP Depotentiation

#### Inhibition of 5α-Reductase by Finasteride Prevents the LFS-DP

**Figure 3:**
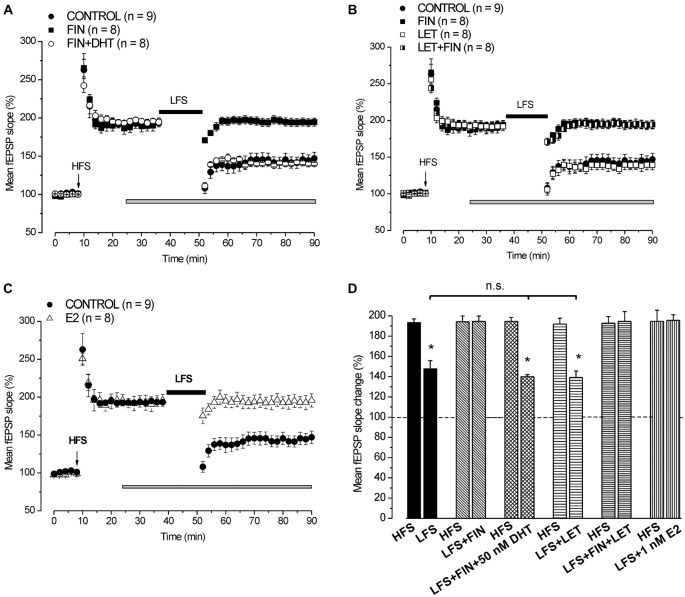
**Neo-synthesis of DHT is involved in the induction of DP by LFS. (A)** Effect of LFS in control condition (filled circles), under FIN (filled squares) and FIN + 50 nM DHT (open circles). **(B)** Effects of LFS in control condition (filled circles), under FIN (filled squares), LET (open squares) and FIN + LET (half-filled squares). **(C)** Effect of LFS in control condition (filled circles) and in the presence of 1 nM E2 (open triangles). The drugs were applied 15 min after the induction of HFS-LTP and LFS was delivered 30 min after HFS. In this and following figure the arrows indicate the HFS delivering time. **(D)** Comparison between HFS-LTP and LFS-DP in different conditions as shown in **(A–C)** (Student’s *t* test, *p < 0.05). The LFS-DP values in control condition and under different drugs are compared (one-way ANOVA, *F*_2,22_ = 0.049, *p* = 0.95). Note that the synthesis of DHT, but not that of E2, is required for the induction of LFS-DP, while exogenous E2 is able to prevent the LFS-DP.

In control condition HFS induced LTP (193.4 ± 3.5%, *n* = 9, 4 animals) and LFS delivered 30 min after induced DP reducing LTP to 147.8 ± 8% (*n* = 9, Student’s *t* test, *p* = 0.003; Figure 3). Application of finasteride starting 15 min after HFS had no effect on the already settled LTP (pre-drug 192.4 ± 5.7% vs. post-drug 194.1 ± 5.8%, *n* = 8, 4 animals, Student’s *t* test, *p* = 0.85, Figures 3A,B), but it prevented the LFS-DP (pre-LFS 194.1 ± 5.8% vs. post-LFS 194.5 ± 5.3%, *n* = 8, Student’s *t* test, *p* = 0.82, Figures 3A,B,D).

Administration of 50 nM DHT under finasteride did not affect the induced LTP (pre-drug 194.1 ± 3.1% vs. post-drug 194.6 ± 3.5%, *n* = 8, 3 animals, Student’s *t* test, *p* = 0.8, Figure 3A), but it was able to rescue the LFS-DP (pre-LFS 194.6 ± 3.9% vs. post-LFS 140 ± 1.9%, *n* = 8, Student’s *t* test, *p* < 0.0001; Tukey’s *post hoc* test: LFS + DHT + FIN vs. LFS control, *p* = 0.99, Figures 3A,D). On the whole, this result provides evidence that the DHT neo-synthesis plays a crucial role in the development of LFS-DP.

#### Inhibition of P450-Aromatase by Letrozole does not Affect the Development of LFS-DP

We also assayed the effects of letrozole alone and finasteride plus letrozole on the induction of LFS-DP. Letrozole did not modify the LTP once induced by HFS (pre-drug 193.8 ± 6.1% vs. post-drug 191.9 ± 5.8%, *n* = 8, 3 animals, Student’s *t* test, *p* = 0.37, Figure 3B), or the DP after LFS (pre-LFS 191.9 ± 5.8% vs. post-LFS 139.1 ± 6.4%, *n* = 8, Student’s *t* test, *p* < 0.001, Figure 3B, Tukey’s *post hoc* test: LFS + LET vs. LFS control, *p* = 0.99, Figure 3D) and it did not rescue the DP when suppressed by finasteride (pre-LFS 192.7 ± 6.6% vs. post-LFS 194.5 ± 6%, *n* = 8, 3 animals, Student’s *t* test, *p* = 0.61, Figures 3B,D). Like observed for the LFS-LTD, these results further exclude a role of E2 neo-synthesis in the depressant effects induced by LFS.

#### Exogenous E2 Prevents the LFS-DP

We assayed whether exogenous E2 influenced the induction of LFS-DP. Application of 1 nM E2 starting 15 min after the induction of HFS-LTP had no effect on LTP (pre-drug 196.2 ± 9.6% vs. post-drug 195.6 ± 7.8%, *n* = 8, 3 animals, Student’s *t* test, *p* = 0.82, Figure 3C), but it prevented the LFS-DP (pre-LFS 195.6 ± 7.8% vs. post-LFS 195.6 ± 8.7%, *n* = 8, Student’s *t* test, *p* = 0.98, Figures 3C,D). This finding demonstrates that although the E2 neo-synthesis does not play any role in the LFS-DP, exogenous E2 is able to prevent it.

### Role of E2 and DHT Neo-Synthesis in the Development of HFS-LTP

#### Effect of P450-Aromatase and 5α-Reductase Blockade on the Development of HFS-LTP

**Figure 4:**
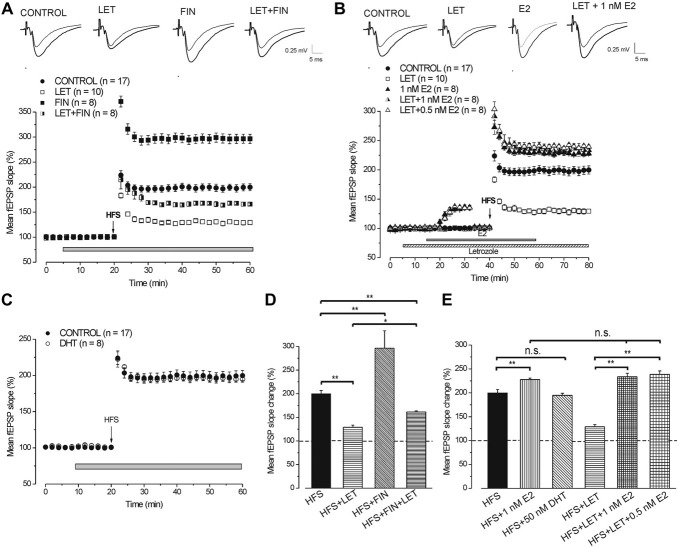
**Neo-synthesis of E2 is involved in the full development of HFS-LTP. (A,B)** On the top averaged traces (*n* = 20) of fEPSPs recorded before (thin traces) and 40 min after HFS (thick traces) in different experimental conditions. **(A)** Effect of HFS in control condition (filled circles), under LET (open squares), FIN (filled squares) and LET + FIN (half-filled squares). **(B)** Effect of HFS in control condition (filled circles), in the presence of LET (open squares), 1 nM E2 (filled triangles), LET + 1 nM E2 (half-filled triangles) and LET + 0.5 nM E2 (open triangles). **(C)** Effect of HFS in control condition (filled circles) and in the presence of 50 nM DHT (open circles). **(D)** Comparison among LTP induced by HFS in control condition and under blockade of E2 and DHT synthesis as shown in **(A)** (one-way ANOVA, *F*_3,39_ = 87.7, *p* < 0.0001; Tukey’s *post hoc* test **p < 0.001, *p < 0.05). **(E)** Comparison among LTP induced by HFS in control condition, under exogenous DHT and E2 with or without LET (one-way ANOVA, *F*_5,54_ = 42.6, *p* < 0.0001; Tukey’s *post hoc* test **p < 0.001). Note that for the full development of HFS-LTP the synthesis of E2 is mainly requested and the block of DHT synthesis further increases LTP.

In full agreement with our previous results (Grassi et al., 2011), HFS in the presence of letrozole induced LTP (129 ± 4.3%, *n* = 10, 4 animals, Figures 4A,B) that was significantly smaller than that obtained in the control condition (199.8 ± 7%, *n* = 17, 6 animals, Tukey’s *post hoc* test: HFS + LET vs. HFS control, *p* < 0.001, Figure 4D). By contrast, LTP induced under finasteride was markedly enhanced (296.5 ± 9.1%, *n* = 8, 3 animals, Tukey’s *post hoc* test: HFS + FIN vs. HFS control, *p* < 0.001, Figures 4A,D). In addition, the combined application of finasteride and letrozole caused LTP (165.3 ± 2.2%, *n* = 8, 3 animals, Figure 4A) of an amplitude that was in-between the control LTP and LTP under letrozole alone (Tukey’s *post hoc* test: HFS + LET + FIN vs. HFS control, *p* < 0.01, HFS + LET + FIN vs. HFS + LET, *p* < 0.01, Figures 4A,D).

These results demonstrate that the blockade of E2 and DHT synthesis remarkably alters the response to HFS by decreasing or increasing the amplitude of LTP, respectively.

#### Exogenous E2 Rescues LTP When HFS is Delivered Under the P450-Aromatase Inhibitor Letrozole

Application of exogenous 1 nM E2 increased the baseline fEPSP (136.1 ± 3.4%, *n* = 8, 3 animals, Figure 4B) and the HFS-LTP to 227.8 ± 3.2% (*n* = 8, Figure 4B) a value that was higher than that observed in the control condition (Tukey’s *post hoc* test: HFS+1 nM E2 vs. HFS control, *p* < 0.05, Figure 4E). This enhancement of LTP was observed after reducing the stimulus intensity to cancel the baseline increase.

Similarly, application of 1 nM E2 in the presence of letrozole enhanced the baseline (135.2 ± 3.8%, *n* = 8, 4 animals, Figure 4B) and rescued the HFS-LTP to a value (233.8 ± 7.3%, *n* = 8) that was not different from that observed after HFS under E2 alone (Tukey’s *post hoc* test: HFS + LET + 1 nM E2 vs. HFS + 1 nM E2, *p* = 0.98, HFS + LET + 1 nM E2 vs. HFS + LET, *p* < 0.001, Figures 4B,E). In addition, no difference was observed by using E2 at lower concentration (0.5 nM), either on the baseline (135.2 ± 2.4%, *n* = 8, 3 animals, Figure 4B) or on the HFS-LTP (239.6 ± 2.8%, *n* = 8, Tukey’s *post hoc* test: LET + 0.5 nM E2 vs. LET + 1 nM E2, *p* = 0.98, Figures 4B,E).

#### Exogenous DHT does not Influence the HFS-LTP

We verified whether exogenous DHT might influence the development of LTP by delivering HFS in the presence of 50 nM DHT. DHT did not interfere with the HFS-LTP (194.7 ± 4.2%, *n* = 8, 3 animals, Tukey’s *post hoc* test: HFS + 50 nM DHT vs. HFS control, *p* = 0.99, Figures 4C,E).

### Different Involvement of DHT and E2 Neo-Synthesis in LTP Induced by WFS and MFS

**Figure 5:**
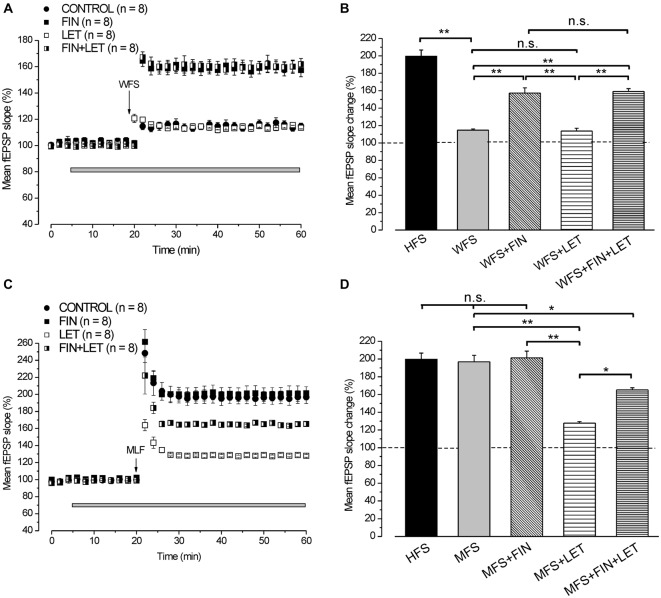
**LTP induced by WFS (1 s-25 Hz) and MFS (1 s-50 Hz). (A)** Effect of WFS in control condition (filled circles), in the presence of FIN (filled squares), LET (open squares) and FIN + LET (half-filled squares). **(B)** Comparison among LTP induced by HFS in control condition and by WFS in the different conditions as shown in A (one-way ANOVA, *F*_4,44_ = 42.25, *p* < 0.0001; Tukey’s *post hoc* test **p < 0.001). **(C)** Effect of MFS in control condition (filled circles), in the presence of FIN (filled squares), LET (open squares) and FIN + LET (half-filled squares). **(D)** Comparison among LTP induced by HFS in control condition and by MFS in the different conditions as shown in C (one-way ANOVA, *F*_4,43_ = 16.76, *p* < 0.0001; Tukey’s *post hoc* **p < 0.001, *p < 0.05). Note that the synthesis of DHT is mainly implied in the WFS induced LTP, while that of E2 is only involved in LTP induced by MFS.

By using WFS (1 s-25 Hz) a very small LTP was induced (114.8 ± 2.2%, *n* = 8, 4 animals, Figure 5A) compared with that induced by HFS (Tukey’s *post hoc* test: WFS control vs. HFS control, *p* < 0.001, Figure 5B). The contribution of the neo-synthesis of DHT and/or E2 in the induction of this small LTP was examined by applying finasteride, letrozole or finasteride plus letrozole. In the presence of finasteride WFS induced a robust LTP (157.4 ± 5.9%, *n* = 8, 3 animals, Tukey’s *post hoc* test: WFS + FIN vs. WFS control, *p* < 0.001, Figures 5A,B), while in the presence of letrozole LTP was not different from the control one (113.7 ± 2%, *n* = 8, 3 animals, Tukey’s *post hoc* test: WFS + LET vs. WFS control, *p* = 0.99, WFS + LET vs. WFS + FIN, *p* < 0.001, Figures 5A,B). The addition of letrozole to finasteride did not modify the amplitude of LTP compared to that induced under finasteride alone (162 ± 4.2%, *n* = 8, 3 animals, Tukey’s *post hoc* test: WFS + FIN + LET vs. WFS + FIN, *p* = 0.98, WFS + FIN + LET vs. WFS + LET, *p* < 0.001, WFS + FIN + LET vs. WFS control, *p* < 0.001, Figures 5A,B). Conversely, MLF (1 s-50 Hz) induced LTP (196.8 ± 7.4%, *n* = 8, 4 animals, Figure 5C) that was not different from that induced by HFS (Tukey’s *post hoc* test: MFS control vs. HFS control, *p* = 0.99, Figure 5D). This LTP was significantly reduced in the presence of letrozole (127.9 ± 1.6%, *n* = 8, 3 animals, Tukey’s *post hoc* test: MFS + LET vs. MFS control, *p* < 0.001, Figures 5C,D), but it was not changed under finasteride (201.3 ± 7.7%, *n* = 8, 3 animals, Tukey’s *post hoc* test: MFS + FIN vs. MFS control, *p* = 0.95, Figures 5C,D). In addition, under combined application of letrozole and finasteride the amplitude of MLF-LTP (165.2 ± 2.4, *n* = 8, 3 animals, Figure 5C) was in-between the control and letrozole values (Tukey’s *post hoc* test: MFS + FIN + LET vs. MFS control, *p* < 0.05, MFS + FIN + LET vs. MFS + LET, *p* < 0.01, Figure 5D). These results suggest that for the WFS long-term response the synthesis of DHT is involved, whereas E2 does not play any role. Conversely, the synthesis of E2 is only required for the development of a full LTP by MFS.

### Comparison of the Responses Observed Under Blockade of E2 (Letrozole) and E2 Plus DHT (Letrozole Plus Finasteride) Syntheses Across Different Stimulation Protocols

**Figure 6:**
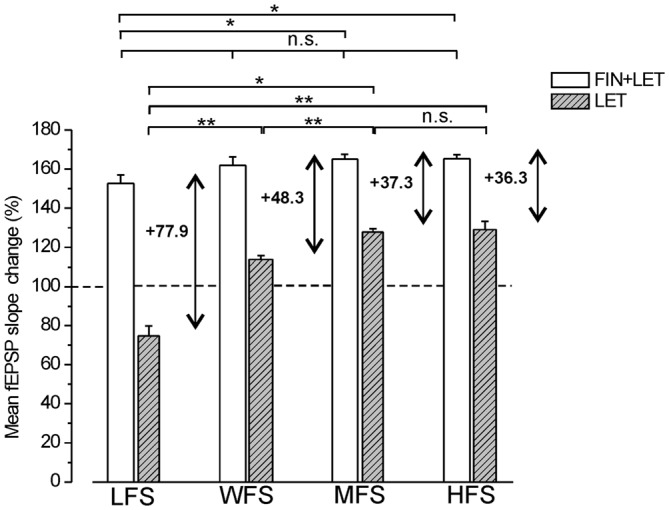
**Comparison of the responses induced by different stimulation patterns under combined blockade of E2 and DHT synthesis and under block of E2 alone.** The amplitude of the responses induced by LFS, WFS, MFS and HFS are compared under FIN + LET (white columns, one-way ANOVA, *F*_3,27_ = 3.39, Tukey’s *post hoc* test *p < 0.05) and under LET alone (hatched gray columns, one-way ANOVA, *F*_3,30_ = 50.34, *p* < 0.0001, Tukey’s *post hoc* test **p < 0.001, *p < 0.05). The graph also reports the difference between the values observed under combined and single block (two headed arrow and number) for revealing the inhibitory contribution of DHT at the different stimulation frequencies. Note the progressive decrease of the DHT influence.

The partial LTPs observed under combined blockade of E2 and DHT synthesis were compared across all different stimulus protocols (LFS, MFS, WFS and HFS). A small enhancement of LTP amplitude appeared at higher frequencies, but the differences were significant only between LFS-LTP and LTP induced by MFS and HFS (Tukey’s *post hoc* test: LFS vs. WFS, *p* = 0.1, LFS vs. MFS, *p* < 0.05, LFS vs. HFS, *p* < 0.05, WFS vs. MFS, *p* = 0.93, WFS vs. HFS, *p* = 0.83, MFS vs. HFS, *p* = 0.99, Figure 6). Moreover, the responses under blockade of E2 synthesis alone significantly changed passing from full LTD by LFS to an increasing LTP at WFS, MFS and HFS (Tukey’s *post hoc* test: LFS vs. WFS, *p* < 0.001, LFS vs. MFS, *p* < 0.001, LFS vs. HFS, *p* < 0.001, WFS vs. MFS, *p* < 0.05, WFS vs. HFS, *p* < 0.05, MFS vs. HFS, *p* = 0.99, Figure 6).

The comparison of the responses observed under combined and single block across all stimulation patterns allows the evaluation of the inhibitory effect of the DHT at different stimulation frequencies. The DHT inhibition was indeed computable by subtracting the amplitude of the responses under letrozole from that under finasteride plus letrozole. The inhibitory influence, as resulting from this computation, was present throughout all stimulus patterns, but diminished progressively by increasing the stimulus frequency (LFS: 77.8%, WFS: 48.3%, MFS: 37.3%, HFS: 36.3%, Figure 6). In fact, the amplitude of responses under letrozole significantly increased showing a large change passing from LFS to HFS, while the partial LTP obtained under combined block of E2 and DHT showed a minor change that was significant only between LFS and MFS or HFS (Figure 6).

## Discussion

This study demonstrates that the neo-syntheses of DHT and E2 during synaptic stimulation are crucial for the development and the sign of the long-term synaptic modification in the hippocampus CA1 region of male rat. In fact, the LTD and DP induced by LFS were prevented by finasteride, a blocking agent for the 5α-reductase enzyme converting T into DHT, while LTP induced by HFS was markedly reduced by letrozole, an inhibitor of the P450-aromatase mediating conversion of T into E2.

**Figure 7:**
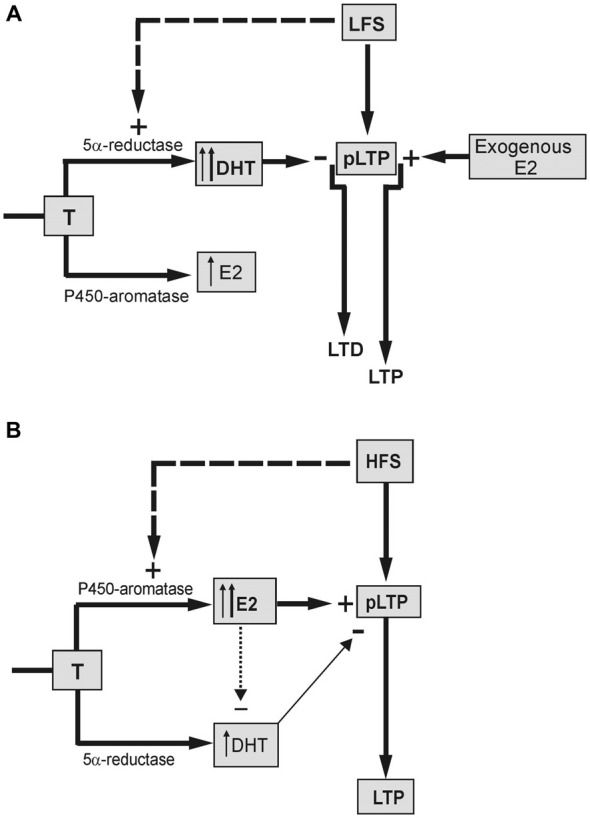
**Schematic draw showing the different influence of neo-synthesized DHT and E2 on the LFS-LTD and HFS-LTP. (A)** LTD is induced in the presence of basal DHT synthesized by 5α-reductase enzyme from T (thin up arrow) and/or DHT synthesized by LFS (dashed arrow, thick up arrow). DHT exerts an inhibitory effect reverting the basal partial LTP (pLTP) into LTD. Synthesis of E2 is not involved in the LFS-LTD. but the exogenous E2 can enhance the pLTP to a full LTP. **(B)** pLTP is changed into a full LTP in the presence of basal E2 synthesized from T by P450-aromatase (thin up arrow) and/or E2 synthesized by HFS (dashed arrow, thick up arrow). The minor inhibitory effect of DHT on pLTP (thin arrow) might be prevented by an inhibitory influence of E2 on DHT (dotted arrow).

About the long-term effects of LFS, LTD induced in naïve neurons was fully prevented by finasteride, and a small LTP, instead, was evoked (Figure 7A). In addition, finasteride precluded the LFS-DP in neurons previously potentiated by HFS. The role of DHT synthesis was confirmed by the rescue of LTD and DP yielded by high concentration (50 nM) of DHT administered in the presence of finasteride, while lower concentration (10 nM) was inefficacious.

Considering that the basal synaptic activity was not influenced by exogenous DHT, we conclude that the block of DHT neo-synthesis definitely affects LTD and DP by interacting with their induction mechanism. Conversely, the synthesis of DHT was not implied in the maintenance of LTD since finasteride did not modify LTD, once settled.

Actually, the 5α-reductase is also implied in the synthesis of other neurosteroids, like the tetrahydrodeoxycorticosterone (THDOC) and allopregnanolone (ALLO) that are facilitatory allosteric modulators of GABA_A_ receptors (Lambert et al., 2003; Reddy, 2003). However, the androgenic neurosteroids have certainly a determinant role in the induction of LTD and DP since these long-term synaptic changes produced by LFS were also fully prevented by the blockade of ARs (Pettorossi et al., 2013). For the same reason, we exclude the influence of the DHT metabolites such as Adiol, which is reported to interfere with GABAA-mediated GABAergic transmission (Frye et al., 2001; Edinger et al., 2004; Reddy, 2004; Reddy and Jian, 2010).

Conversely, since ARs can bind both DHT and T, the involvement of T in LTD might be proposed. However, the finding that exogenously administered T, differently from DHT, was not able to rescue the LTD under finasteride, excludes T participation. To justify this diverse influence we took into account the lower receptor affinity of T compared with DHT (Zhou et al., 1995; Fang et al., 2003), which might also explain why the upstream accumulation of T, occurring under block of DHT synthesis, was not able to substitute for the lack of DHT.

We also examined the possible role of the neo-synthesis of E2 in the LFS depressive responses by applying letrozole. Both LFS-LTD and DP were not affected by letrozole, suggesting that E2 has no influence in the induction of these phenomena. In addition, under letrozole, we did not observe any enhancement of LTD and DP, as expected in the case of increase of DHT synthesis due to upstream accumulation of T. This suggests that the DHT-dependent LFS effects are not influenced by additional DHT, as also supported by the inability of exogenous DHT to modify the LFS-LTD.

Although E2 is not implied in the LFS-LTD, administration of E2 was indeed able to revert LTD into a robust LTP (Figure 7A). This suggests that the plastic events induced by LFS may be influenced by E2, but this influence is not normally possible due to a too low level of basally synthesized E2 and/or the stimulus inability to increase it. Unlike LTD and DP, E2 neo-synthesis is relevant for the full induction of the LTP (Grassi et al., 2011; Tanaka and Sokabe, 2012; Figure 7B), while it does not influence LTP maintenance (Grassi et al., 2011). Here, we found that under blockade of P450-aromatase the application of 1 or 0.5 nM E2 fully rescued LTP, enhancing its amplitude to values even higher than the control one. This rescue was not due to normal increase of the baseline induced by exogenous E2 since similar LTP was observed after lowering the stimulus intensity to recover the pre-E2 baseline.

Another point of interest concerns the role of DHT in HFS-LTP. Blockade of DHT synthesis during HFS induced a remarkable enhancement of the LTP amplitude. Is this evidence for a persistent influence of an inhibitory role of DHT at HFS, or is it the result of enhanced synthesis of E2 due to upstream accumulation of T? The fact that, differently from finasteride, AR antagonism did not change the amplitude of HFS-LTP (Pettorossi et al., 2013) straightforwardly supports the effect of T accumulation. However, the LTP obtained under combined block of E2 and DHT synthesis was higher than that observed under letrozole alone, suggesting the presence of an inhibitory DHT influence. It is likely that this influence is prevented or masked when the E2 synthesis is allowed (Figure 7B).

The contribution of E2 and DHT has been also examined on the responses induced by intermediate stimulation frequencies: WFS (1 s-25 Hz) and MFS (1 s-50 Hz). WFS elicited a small LTP that was enhanced by finasteride, but unaffected by letrozole, as occurs for the LFS-LTD. Conversely, MFS elicited a LTP that was similar to that induced by HFS and similarly was reduced by letrozole, but, at variance, not enhanced by finasteride.

This different effect might be due to the inability of MFS to drive a further synthesis of E2 from the T accumulated following the block of DHT synthesis. These results support the idea of a frequency dependent T-E2 conversion that is less powerful or null at lower frequencies.

On the whole, a basal partial LTP is only inducible, independently of stimulation pattern (LFS, WFS, MFS and HFS), under combined blockade of E2 and DHT synthesis. It is, in fact, the synthesis of E2 responsible for the enhancement of this basal LTP to a full LTP following MFS and HFS, and that of DHT that reverts the LTP into LTD following LFS. In contrast, with the influence of E2 that is only limited to the range of higher stimulation frequencies, DHT is maximally operative in the range of low frequencies, but its effect persists, even if with minor extent, across all tested frequencies. The amount of the DHT effect is detectable by computing the difference between the amplitude of LTP under combined blocks and under block of E2 synthesis alone.

The reason for a frequency dependent differential effect of E2 and DHT might be related to the specific interaction between the stimulus frequency and the basally synthesized neurosteroids, or to a specific capability of the stimulation patterns to increase neurosteroid synthesis depending on their frequency (Figure 7).

The frequency dependent activation of P450-aromatase and 5α-reductase on synaptic transmission is conceivable since different levels of Ca^2+^ entry modulated by the nervous activity (Kimoto et al., 2001; Balthazart and Ball, 2006; Hojo et al., 2008) may influence the enzymatic function. It is known, in fact, that LTP or LTD is driven by different velocity and amount of NMDAR-mediated Ca^2+^ increase (Dudek and Bear, 1992; Bear and Malenka, 1994; Cummings et al., 1996). In line with this evidence, we suggest that while DHT synthesis, even if it seems to be activated by a broad range of frequencies, is mostly enhanced by low frequency inducing a low Ca^2+^ entry (Dudek and Bear, 1992; Bear and Malenka, 1994; Cummings et al., 1996), E2 synthesis is triggered by HFS inducing high Ca^2+^ entry. However, the possible enzymatic activation of P450-aromatase by high Ca^2+^ entry is in contrast with data in the literature. In fact, a rapid inhibition of P450-aromatase, via a Ca^2+^-dependent phosphorylation, has been evidenced following increases of intracellular Ca^2+^ due to K^+^-induced depolarization or activation of glutamatergic receptors (Balthazart et al., 2001, 2003, 2006; Charlier et al., 2015). This prompts for a reduction of E2 synthesis during HFS. Conversely, our study suggests that the increase of E2 neo-synthesis should be driven by HFS, while increase of DHT neo-synthesis by LFS. To overcome this divergence, we propose that the velocity of Ca^2+^ entry following synaptic activation is a crucial point influencing differently phosphorilation-dephosphorilation processes for activating P450-aromatase or 5α-reductase enzymes.

Concerning the mechanisms through which new synthesized estrogenic and androgenic neurosteroids lead to long-term synaptic changes, we suggest that the activation of ERs and ARs might produce a functional up- or down-regulation of the NMDARs, respectively (Pouliot et al., 1996; Foy et al., 1999; Smith and McMahon, 2005, 2006; Grassi et al., 2010) and influence the GABAergic neurotransmission in opposite ways, by interacting with the GABARs (Murphy et al., 1998; Frye et al., 2001; Rudick and Wooley, 2001; Edinger et al., 2004).

The influence of androgenic and estrogenic signals is probably exerted at postsynaptic level, as blockade of either ARs or ERs did not affect the facilitated responses to paired stimuli (Pettorossi et al., 2013).

Despite the need of further insight of the site and mechanism of neurosteroids in the synaptic plasticity, the current study puts forward a crucial function of neo-synthesized E2 and DHT in the induction and direction of the hippocampal synaptic plasticity.

Since our study has been performed only in male rat, we should be cautious in generalizing these mechanisms as it may vary depending on sex, estrous cycle and age, as shown in the vestibular system (Pettorossi et al., 2011; Grassi et al., 2012).

However, in this work we definitely demonstrated that specific stimulation patterns within the CNS are able to determine the amplitude and the sign of long-term synaptic effects through the neo-synthesis of E2 or DHT. Neural E2 and DHT should thus be recognized as very effective central modulators of synaptic plasticity that may significantly contribute to learning and memory performance.

## Author Contributions

The experiments were performed in the laboratory of SG at the University of Perugia. VEP, SG and PC designed the experiments and wrote the manuscript; MDM and AT performed and analyzed the experiments. All authors provided important intellectual content and critically revised the final version of the manuscript.

## Funding

This work was supported by grants from Progetto di Ricerca di Interesse Nazionale (PRIN) 2010-2011 AHHP5H to PC and SG and Fondazione Cassa di Risparmio di Perugia (2009.020.0036 to PC and VEP and 2014-0104 to AT).

## Conflict of Interest Statement

PC is a member of the editorial boards of Lancet Neurology, Journal of Neuroscience, Movement Disorders, and Synapse and receives research support from Biogen, Lundbeck, Merck-Serono, Sanofi-Aventis, UCB, Fondazione Santa Lucia IRCCS and Ministero della Salute. The other authors declare that the research was conducted in the absence of any commercial or financial relationships that could be construed as a potential conflict of interest.

## Abbreviations

ARs: androgen receptors
DHT: 5α-dihydrotestosterone
DP: depotentiation
E2: 17b-estradiol
ERs: estrogen receptors
fEPSP: field excitatory postsynaptic potential
FIN: finasteride
HFS: high frequency stimulation
LET: letrozole
LFS: low frequency stimulation
LTD: long-term depression
LTP: long-term potentiation
MFS: medium frequency stimulation
NMDAR: NMDA receptors
T: testosterone
WFS: weak frequency stimulation.

## References

[B1] Balthazart J. Ball G. F. (2006). Is brain estradiol a hormone or a neurotransmitter? Trends Neurosci. 29, 241–249. 10.1016/j.tins.2006.03.004 16580076

[B2] Balthazart J. Baillien M. Ball G. F. (2001). Phosphorylation processes mediate rapid changes of brain aromatase activity. J. Steroid Biochem. Mol. Biol. 79, 261–277. Neuroendocrinol.26,63–73. 10.1016/s0960-0760(01)00143-1 11850233

[B3] Balthazart J. Baillien M. Ball G. F. (2006). Rapid control of brain aromatase activity by glutamatergic inputs. Endocrinology 147, 359–366. 10.1210/en.2005-0845 16195408

[B4] Balthazart J. Baillien M. Charlier T. D. Ball G. F. (2003). Calcium-dependent phosphorylation processes control brain aromatase in quail. Eur. J. Neurosci. 17, 1591–1606. 10.1046/j.1460-9568.2003.02598.x 12752377

[B5] Baulieu E. E. (1997). Neurosteroids: of the nervous system, by the nervous system, for the nervous system. Rec. Prog. Horm. Res. 52, 1–32. 9238846

[B6] Bear M. F. Malenka R. C. (1994). Synaptic plasticity: LTP and LTD. Curr. Opin. Neurobiol. 4, 389–399. 10.1016/0959-4388(94)90101-5 7919934

[B7] Bhatnagar A. S. Brodie A. M. H. Long B. J. Evans D. B. Miller W. R. (2001). Intracellular aromatase and its relevance to the pharmacological efficacy of aromatase inhibitors. J. Steroid Biochem. Mol. Biol. 76, 199–202. 10.1016/s0960-0760(01)00050-4 11384878

[B8] Bi R. Foy M. R. Vouimba R. M. Thompson R. F. Baudry M. (2001). Cyclic changes in estradiol regulate synaptic plasticity through the MAP kinase pathway. Proc. Natl. Acad. Sci. U S A 98, 13391–13395. 10.1073/pnas.241507698 11687663 PMC60881

[B9] Bliss T. V. P. Collingridge G. L. (1993). A synaptic model of memory: long-term potentiation in the hippocampus. Nature 361, 31–39. 10.1038/361031a0 8421494

[B10] Bliss T. V. P. Lomo T. (1973). Long-lasting potentiation of synaptic transmission in the dentate area of the anaesthetized rabbit following stimulation of the perforant path. J. Physiol. 232, 331–356. 10.1113/jphysiol.1973.sp010273 4727084 PMC1350458

[B11] Charlier T. D. Cornil C. A. Patte-Mensah C. Meyer L. Mensah-Nyagan A. G. Balthazar J. (2015). Local modulation of steroid action: rapid control of enzymatic activity. Front. Neurosci. 9:83. 10.3389/fnins.2015.00083 25852459 PMC4365721

[B12] Compagnone N. A. Mellon S. H. (2000). Neurosteroids: biosynthesis and function of these novel neuromodulators. Front. Neuroendocrinol. 21, 1–56. 10.1006/frne.1999.0188 10662535

[B13] Cummings J. A. Mulkey R. M. Nicoll R. A. Malenka R. C. (1996). Ca2+ signalling requirements for long-term depression in the hippocampus. Neuron 16, 825–833. 10.1016/s0896-6273(00)80102-6 8608000

[B14] Dudek S. M. Bear M. F. (1992). Homosynaptic long-term depression in area CA1 of hippocampus and effects of N-methyl-D-aspartate receptor blockade. Proc. Natl. Acad. Sci. U S A 89, 4363–4367. 10.1073/pnas.89.10.4363 1350090 PMC49082

[B15] Dudek S. M. Bear M. F. (1993). Bidirectional long-term modification of synaptic effectiveness in the adult and immature hippocampus. J. Neurosci. 13, 2910–2918. 8331379 10.1523/JNEUROSCI.13-07-02910.1993 PMC6576673

[B16] Edinger K. L. Lee B. Frye C. A. (2004). Mnemonic effects of testosterone and its 5*α*-reduced metabolites in the conditioned fear and inhibitory avoidance tasks. Pharmacol. Biochem. Behav. 78, 559–568. 10.1016/j.pbb.2004.04.024 15251265

[B17] Fang H. Tong W. Branham W. S. Moland C. L. Dial S. L. Hong H.. (2003). Study of 202 natural, synthetic and environmental chemicals for binding to the androgen receptor. Chem. Res. Toxicol. 16, 1338–1358. 10.1021/tx030011g 14565775

[B18] Finn D. A. Beadles-Bohling A. S. Beckley E. H. Ford M. M. Gilliland K. R. Gorin-Mneyer R. E.. (2006). A new look at the 5alpha-reductase inhibitor finasteride. CNS Drug Rev. 12, 53–76. 10.1111/j.1527-3458.2006.00053.x 16834758 PMC6741762

[B19] Foradori C. D. Weiser M. J. Handa R. J. (2008). Non-genomic actions of androgens. Front. Neuroendocrinol. 29, 169–181. 10.1016/j.yfrne.2007.10.005 18093638 PMC2386261

[B20] Foy M. R. (2001). 17beta-estradiol: effects on CA1 hippocampal synaptic plasticity. Neurobiol. Learn. Mem. 76, 239–252. 10.1006/nlme.2001.4018 11726235

[B21] Foy M. R. Xu J. Xie X. Brinton R. D. Thompson R. F. Berger T. W. (1999). 17beta-Estradiol enhances NMDA receptor-mediated EPSPs and long-term potentiation. J. Neurophysiol. 81, 925–929. 10036289 10.1152/jn.1999.81.2.925

[B22] Frye C. A. Park D. Tanaka M. Rosellini R. Svare B. (2001). The testosterone metabolite and neurosteroid 3alpha-androstanediol may mediate the effects of testosterone on conditioned place preference. Psychoneuroendocrinology 26, 731–750. 10.1016/s0306-4530(01)00027-0 11500254

[B23] Good M. Day M. Muir J. L. (1999). Cyclical changes in endogenous levels of oestrogen modulate the induction of LTD and LTP in the hippocampal CA1 region. Eur. J. Neurosci. 11, 4476–4480. 10.1046/j.1460-9568.1999.00920.x 10594677

[B24] Grassi S. Frondaroli A. Dieni C. Scarduzio M. Pettorossi V. E. (2009). Long-term potentiation in the rat medial vestibular nuclei depends on locally synthesised 17beta-Estradiol. J. Neurosci. 29, 10779–10783. 10.1523/JNEUROSCI.1697-09.2009 19710328 PMC6665688

[B25] Grassi S. Frondaroli A. Scarduzio M. Dieni C. V. Brecchia G. Boiti C.. (2012). Influence of sex and estrous cycle on synaptic responses of the medial vestibular nuclei in rats: role of circulating 17*β*-estradiol. Brain Res. Bull. 87, 319–327. 10.1016/j.brainresbull.2011.11.008 22127323

[B26] Grassi S. Frondaroli A. Scarduzio M. Dutia B. Dieni C. Pettorossi V. E. (2010). Effects of 17b-estradiol on glutamate synaptic transmission and neuronal excitability in the rat medial vestibular nuclei. Neuroscience 165, 1100–1114. 10.1016/j.neuroscience.2009.11.039 19944747

[B27] Grassi S. Tozzi A. Costa C. Tantucci M. Colcelli E. Scarduzio M.. (2011). Neural 17beta-Estradiol facilitates long-term potentiation in the hippocampal CA1 region. Neuroscience 192, 67–73. 10.1016/j.neuroscience.2011.06.078 21749911

[B28] Hajszan T. MacLusky N. J. Leranth C. (2008). Role of androgens and the androgen receptor in remodeling of spine synapses in limbic brain areas. Horm. Behav. 53, 638–646. 10.1016/j.yhbeh.2007.12.007 18262185 PMC2408746

[B29] Harley C. W. Malsbury C. W. Squires A. Brown R. A. M. (2000). Testosterone decreases CA1 plasticity in vivo in gonadectomized male rats. Hippocampus 10, 693–696. 10.1002/1098-1063(2000)10:6<693::aid-hipo1007>3.0.co;2-g 11153715

[B30] Hasegawa Y. Hojo Y. Kojima H. Ikeda M. Hotta K. Sato R.. (2015). Estradiol rapidly modulates synaptic plasticity of hippocampal neurons: involvement of kinase networks. Brain Res. 1621, 147–161. 10.1016/j.brainres.2014.12.056 25595055

[B31] Hebbard P. C. King R. R. Malsury C. W. Harley C. W. (2003). Two organizational effects of pubertal testosterone in male rats: transient social memory and a shift away from long-term potentiation following a tetanus in hippocampal CA1. Exp. Neurol. 182, 470–475. 10.1016/s0014-4886(03)00119-5 12895458

[B32] Hojo Y. Hattori T. A. Enami T. Furukawa A. Suzuki K. Ishii H. T.. (2004). Adult male rat hippocampus synthesizes estradiol from pregnenolone by cytochromes P450 17alpha and P450 aromatase localized in neurons. Proc. Natl. Acad. Sci. U S A 101, 865–870. 10.1073/pnas.2630225100 14694190 PMC321772

[B33] Hojo Y. Higo S. Ishii H. Ooishi Y. Mukai H. Murakami G.. (2009). Comparison between hippocampus-synthesized and circulation-derived sex steroids in the hippocampus. Endocrinology 150, 5106–5112. 10.1210/en.2009-0305 19589866

[B34] Hojo Y. Murakami G. Mukai H. Higo S. Hatanaka Y. Ikeda M. O.. (2008). Estrogen synthesis in the brain. Role on synaptic plasticity and memory. Mol. Cell. Endocrinol. 290, 31–43. 10.1016/j.mce.2008.04.017 18541362

[B35] Isgor C. Sengelaub D. R. (2003). Effects of neonatal gonadal steroids on adult CA3 pyramidal neuron dendritic morphology and spatial memory in rats. J. Neurobiol. 55, 179–190. 10.1002/neu.10200 12672016

[B36] Kalita K. Szymczak S. Kaczmarek L. (2005). Non-nuclear estrogen receptor beta and alpha in the hippocampus of male and female rats. Hippocampus 15, 404–412. 10.1002/hipo.20066 15669092

[B37] Kerr J. E. Allore R. J. Bech S. G. Hande R. J. (1995). Distribution and hormonal-regulation of androgen receptor (AR) and AR messenger ribonucleic acid in the rat hippocampus. Endocrinology 136, 3213–3221. 10.1210/en.136.8.3213 7628354

[B38] Kimoto T. Tsurugizawa T. Ohta Y. Makino J. Tamura H. Hojo Y.. (2001). Neurosteroid synthesis by citochrome p450-containing systems localized in the rat brain. Endocrinology 142, 3578–3589. 10.1210/en.142.8.3578 11459806

[B39] Kramár E. A. Chen L. Y. Brandon N. J. Rex C. S. Liu F. Gall C. M.. (2009). Cytoskeletal changes underline estrogen’s acute effects on synaptic transmission and plasticity. J. Neurosci. 29, 12982–12993. 10.1523/JNEUROSCI.3059-09.2009 19828812 PMC2806054

[B40] Lambert J. J. Belelli D. Peden D. R. Vardy A. W. Peters J. A. (2003). Neurosteroids modulation of GABAA receptors. Prog. Neurobiol. 71, 67–80. 10.1016/j.pneurobio.2003.09.001 14611869

[B41] Levin E. R. (2009). Plasma membrane estrogen receptors. Trends Endocrinol. Metab. 20, 477–482. 10.1016/j.tem.2009.06.009 19783454 PMC3589572

[B42] MacLusky N. J. Hajszan T. Prange-Kiel J. Leranth C. (2006). Androgen modulation of hippocampal synaptic plasticity. Neuroscience 138, 957–965. 10.1016/j.neuroscience.2005.12.054 16488544

[B43] Malenka R. C. Bear M. F. (2004). LTP and LTD: an embarrassment of riches. Neuron 44, 5–21. 10.1016/j.neuron.2004.09.012 15450156

[B44] Malenka R. C. Nicoll R. A. (1999). Neuroscience-Long-term potentiation-a decade of progress? Science 285, 1870–1874. 10.1126/science.285.5435.1870 10489359

[B45] Martin S. J. Grimwood P. D. Morris R. G. (2000). Synaptic plasticity and memory: an evaluation of the hypothesis. Annu. Rev. Neurosci. 23, 649–711. 10.1146/annurev.neuro.23.1.649 10845078

[B46] McEwen B. S. (2002). Estrogen actions throughout the brain. Recent Prog. Horm. Res. 57, 357–384. 10.1210/rp.57.1.357 12017552

[B47] Milner T. A. Ayoola K. Drake C. T. Herrick S. P. Tabori N. E. McEwen B. S.. (2005). Ultrastructural localization of estrogen receptor beta immunoreactivity in the rat hippocampal formation. J. Comp. Neurol. 491, 81–95. 10.1002/cne.20724 16127691

[B48] Milner T. A. McEwen B. S. Hayashi S. Li C. J. Reagan L. P. Alves S. E. (2001). Ultrastructural evidence that hippocampal alpha estrogen receptors are located at extranuclear sites. J. Comp. Neurol. 429, 355–371. 10.1002/1096-9861(20010115)429:3<355::aid-cne1>3.0.co;2-# 11116225

[B49] Morissette M. Le Saux M. D’Astous M. Jourdain S. Al Sweidi S. Morin N.. (2008). Contribution of estrogen receptors alpha and beta to the effects of estradiol in the brain. J. Steroid Biochem. Mol. Biol. 108, 327–338. 10.1016/j.jsbmb.2007.09.011 17936613

[B50] Mukai H. Takata N. Ishii H. T. Tanabe N. Hojo J. Furukawa A.. (2006). Hippocampal synthesis of estrogens androgens which are paracrine modulators of synaptic plasticity: synaptocrinology. Neuroscience 138, 757–764. 10.1016/j.neuroscience.2005.09.010 16310315

[B51] Mulkey R. M. Malenka R. C. (1992). Mechanisms underlying induction of homosynaptic long-term depression in area CA1 of the hippocampus. Neuron 9, 967–975. 10.1016/0896-6273(92)90248-c 1419003

[B52] Murphy D. D. Cole N. B. Greenberger V. Segal M. (1998). Estradiol increases dendritic spine density by reducing GABA neurotransmission in hippocampal neurons. J. Neurosci. 18, 2550–2559. 9502814 10.1523/JNEUROSCI.18-07-02550.1998 PMC6793090

[B53] Ooishi Y. Kawato S. Hojo Y. Hatanaka Y. Higo S. Murakami G.. (2012). Modulation of synaptic plasticity in the hippocampus by hippocampus-derived estrogen androgen. J. Steroid Biochem. Mol. Biol. 131, 37–51. 10.1016/j.jsbmb.2011.10.004 22075082

[B54] Pedram A. Razandi M. Levi E. R. (2006). Nature of functional estrogen receptors at the plasma membrane. Mol. Endocrinol. 20, 1996–2009. 10.1210/me.2005-0525 16645038

[B55] Pettorossi V. E. Di Mauro M. Scarduzio M. Panichi R. Tozzi A. Calabresi P.. (2013). Modulatory role of androgenic and estrogenic neurosteroids in determining the direction of synaptic plasticity in the CA1 hippocampal region of male rats. Physiol. Rep. 1:e00185. 10.1002/phy2.185 24744863 PMC3970743

[B56] Pettorossi V. E. Frondaroli A. Grassi S. (2011). Cyclic estrogenic fluctuation influences synaptic transmission of the medial vestibular nuclei in female rats. Acta Otolaryngol. 131, 434–439. 10.3109/00016489.2010.536992 21189054

[B57] Pouliot W. A. Handa R. J. Beck S. G. (1996). Androgen modulates N-methyl-D-aspartate-mediated depolarization in CA1 hippocampal pyramidal cells. Synapse 23, 10–19. 10.1002/(sici)1098-2396(199605)23:1<10::aid-syn2>3.0.co;2-k 8723131

[B58] Raz L. Khan M. M. Mahesh V. B. Vadlamudi R. K. Brann D. W. (2008). Rapid estrogen signalling in the brain. Neurosignals 16, 140–153. 10.1159/000111559 18253054

[B59] Reddy D. S. (2003). Is there a physiological role for the neurosteroid THDOC in stress-sensitive conditions? Trends Pharmacol. Sci. 24, 103–106. 10.1016/s0165-6147(03)00023-3 12628349

[B60] Reddy D. S. (2004). Anticonvulsant activity of the testosterone-derived neurosteroid 3alpha-androstanediol. Neuroreport 15, 515–518. 10.1097/00001756-200403010-00026 15094514

[B61] Reddy D. S. Jian K. (2010). The testosterone-derived neurosteroid androstanediol is a positive allosteric modulator of GABA A receptors. J. Pharmacol. Exp. Ther. 334, 1031–1041. 10.1124/jpet.110.169854 20551294 PMC2939675

[B62] Rudick C. N. Wooley C. S. (2001). Estrogen regulates functional inhibition of hippocampal CA1 pyramidal cells in the adult female rat. J. Neurosci. 21, 6532–6543. 11517242 10.1523/JNEUROSCI.21-17-06532.2001 PMC6763095

[B63] Selmanoff M. K. Brodkin L. D. Weiner R. I. Siiteri P. K. (1977). Aromatization and 5alpha-reduction of androgens in discrete hypothalamic and limbic regions of the male and female rat. Endocrinology 101, 841–848. 10.1210/endo-101-3-841 891467

[B64] Skucas V. A. Duffy A. M. Harte-Hargrove L. C. Magagna-Poveda A. Radman T. Chakraborty G.. (2013). Testosterone depletion in adult male rats increases mossy fiber transmission, LTP and sprouting in area CA3 of hippocampus. J. Neurosci. 33, 2338–2355. 10.1523/JNEUROSCI.3857-12.2013 23392664 PMC3711621

[B65] Smith C. C. McMahon L. L. (2005). Estrogen-induced increase in the magnitude of long-term potentiation occurs only when the ratio of NMDA transmission to AMPA transmission is increased. J. Neurosci. 25, 7780–7791. 10.1523/jneurosci.0762-05.2005 16120779 PMC6725261

[B66] Smith C. C. McMahon L. L. (2006). Estrogen-induced increase in the magnitude of long-term potentiation is prevented by blocking NR2B-containing receptors. J. Neurosci. 26, 8517–8522. 10.1523/jneurosci.5279-05.2006 16914677 PMC6674362

[B67] Smith C. C. Vedder L. C. McMahon L. L. (2009). Estradiol and the relationship between dendritic spines, NR2B containing NMDA receptors and the magnitude of long-term potentiation at hippocampal CA3-CA1 synapses. Psychoneuroendocrinology 34(Suppl. 1), S130–S142. 10.1016/j.psyneuen.2009.06.003 19596521 PMC2796081

[B68] Staubli U. V. Ji Z. X. (1996). The induction of homo- vs. hetrosynaptic LTD in area CA1 of hippocampal slices from adult rats. Brain Res. 714, 169–176. 10.1016/0006-8993(95)01523-x 8861622

[B69] Staubli U. Lynch G. (1990). Stable depression of potentiated synaptic responses in the hippocampus with 1-5 Hz stimulation. Brain Res. 513, 113–118. 10.1016/0006-8993(90)91096-y 2350674

[B70] Tabori N. E. Stewart L. S. Znamensky V. Romeo R. D. Alves S. E. McEwen B. S.. (2005). Ultrastructural evidence that androgen receptors are located at extranuclear sites in the rat hippocampal formation. Neuroscience 130, 151–163. 10.1016/j.neuroscience.2004.08.048 15561432

[B71] Tanaka M. Sokabe M. (2012). Continuous de novo synthesis of neurosteroids is required for normal synaptic transmission and plasticity in the dentate gyrus of the rat hippocampus. Neuropharmacology 62, 2373–2387. 10.1016/j.neuropharm.2012.02.007 22365983

[B72] Vierk R. Brandt N. Rune G. M. (2014). Hippocampal estradiol synthesis and its significance for hippocampal synaptic stability in male and female animals. Neuroscience 274, 24–32. 10.1016/j.neuroscience.2014.05.003 24846612

[B73] Vierk R. Glassmeier G. Zhou L. Brandt N. Fester L. Dudzinski D.. (2012). Aromatase inhibition abolishes LTP generation in female but not in male mice. J. Neurosci. 32, 8116–8126. 10.1523/JNEUROSCI.5319-11.2012 22699893 PMC6703647

[B74] Warren S. G. Humphreys A. G. Juraska J. M. Greenough W. T. (1995). LTP varies across the estrous cycle: enhanced synaptic plasticity in proestrus rats. Brain Res. 703, 26–30. 10.1016/0006-8993(95)01059-9 8719612

[B75] Wong M. Moss R. L. (1992). Long-term and short-term electrophysiological effects of estrogen on the synaptic properties of hippocampal CA1 neurons. J. Neurosci. 12, 3217–3225. 1353794 10.1523/JNEUROSCI.12-08-03217.1992 PMC6575649

[B76] Woolley C. S. Weiland N. G. McEwen B. S. Schwartzkroin P. A. (1997). Estradiol increases the sensitivity of hippocampal CA1 pyramidal cells to NMDA receptor-mediated synaptic input: correlation with dendritic spine density. J. Neurosci. 17, 1848–1859. 9030643 10.1523/JNEUROSCI.17-05-01848.1997 PMC6573364

[B77] Zhou Z. X. Lane M. V. Kemppainen J. A. French F. S. Wilson E. M. (1995). Specificity of ligand- dependent androgen receptor stabilization: receptor domain interactions influence ligand dissociation and receptor stability. Mol. Endocrinol. 9, 208–218. 10.1210/me.9.2.208 7776971

